# Emotional attentional capture in children with conduct problems: the role of callous-unemotional traits

**DOI:** 10.3389/fnhum.2014.00570

**Published:** 2014-08-26

**Authors:** Sara Hodsoll, Nilli Lavie, Essi Viding

**Affiliations:** ^1^Developmental Risk and Resilience Unit, Department of Clinical, Educational and Health Psychology, University College LondonLondon, UK; ^2^Institute of Cognitive Neuroscience, University College LondonLondon, UK

**Keywords:** callous-unemotional traits, attentional capture, emotional faces, emotion processing, visual search

## Abstract

**Objective:** Appropriate reactivity to emotional facial expressions, even if these are seen whilst we are engaged in another activity, is critical for successful social interaction. Children with conduct problems (CP) and high levels of callous-unemotional (CU) traits are characterized by blunted reactivity to other people's emotions, while children with CP and low levels of CU traits can over-react to perceived emotional threat. No study to date has compared children with CP and high vs. low levels of CU traits to typically developing (TD) children or each other, using a task that assesses attentional capture by irrelevant emotional faces.

**Method:** All participants performed an attentional capture task in which they were asked to judge the orientation of a single male face that was displayed simultaneously with two female faces. Three types of trials were presented, trials with all neutral faces, trials with an emotional distractor face and trials with an emotional target face. Fifteen boys with CP and high levels of CU traits, 17 boys with CP and low levels of CU traits and 17 age and ability matched TD boys were included in the final study sample.

**Results:** Compared to TD children and children with low levels of CU traits, children with CP and high levels of CU traits showed reduced attentional capture by irrelevant emotional faces.

**Conclusions:** This study is the first to demonstrate a different pattern in emotional attentional capture in children with CP depending on their level of CU traits.

## Introduction

Research suggests that children with conduct problems (CP) are a heterogeneous group that can be delineated based on high vs. low levels of callous-unemotional (CU) traits (Frick and Viding, 2009). Children with CP and high levels of CU traits (CP/HCU) are more genetically vulnerable to develop CP, have more CP, show more severe levels of aggression, and have a poorer prognosis than children with CP who have low levels of CU traits (CP/LCU) (Viding et al., 2005; Frick and Viding, 2009). The current evidence base indicates that CP/HCU display impaired recognition of fearful (and in some cases sad) faces, vocal tones, and body poses, as well as reduced psychophysiological reactivity to distressing and threatening images (Blair, 1999; Blair et al., 2001, 2005; Dadds et al., 2006; Fairchild et al., 2009; Munoz, 2009). In contrast, some studies have reported that children with CP/LCU incorrectly categorize neutral faces as being angry and make hostile attribution biases in vignette-based neutral stories (e.g., Cadesky et al., 2000; Frick et al., 2003; Dadds et al., 2006). Apart from recognition of emotion, it is also important to consider whether CP also involves changes in attention to emotional information. In real life situations we tend to process emotional information alongside other stimuli and it is critical for successful social functioning to react to emotional cues, even if these occur when we are engaged in another activity. Although there is a growing body of research into emotion recognition and attribution in different subgroups of children with CP, very little research has focused on investigating attention to emotion in these children.

Kimonis et al. (2006) used an emotional dot-probe task to examine the associations between aggression, psychopathic (including CU) traits and processing of emotional stimuli in a sample of community-based children. On each trial, children were presented briefly with pairs of images, one above and one below a central fixation cross. Immediately following the image display, a dot-probe (an asterisk) appeared in the previous location of one of the images. The children's task was to indicate whether the dot-probe was located in the top or bottom location. Four different types of image-pairs were used: neutral–neutral (e.g., books); neutral—threat (e.g., an attacking dog); neutral—distress (e.g., a crying child); and neutral—pleasant (e.g., a kitten). In dot-probe paradigms it is thought that a stimulus is preferentially attended to if RT to the dot-probe that replaces the stimulus is facilitated. Kimonis and colleagues found reduced RT facilitation to distress but not to other types of images in children who were high in both psychopathic traits and aggression compared to children with low psychopathic traits and low levels of aggression. A subsequent study by Kimonis and colleagues utilized the dot-probe paradigm with incarcerated youth (Kimonis et al., 2008). Youth who were high on CU and had high levels of aggression were less facilitated by distress images than youth with low levels of CU and aggression; a pattern of findings that was similar to that found in their previous study of community children. A recent study by Sylvers et al. (2011) assessed time taken for emotional faces to break through to conscious awareness during a continuous flash suppression task. Elevated CU trait scores were associated with greater lag times for fearful, and, to a lesser extent, disgusted faces, to break through to conscious awareness relative to neutral faces. This effect was particularly pronounced in children who not only had CU traits, but also high levels of impulsive behavior. Finally, studies by Dadds and colleagues show that while fear recognition deficits can be normalized in children with high CU traits by directing them to focus on the eye-region of the face, under spontaneous viewing conditions these children do not focus on this region of the face as much as typically developing (TD) children do (Dadds et al., 2006, 2008). This suggests that the deficit, in the first place, is in spontaneous orienting to the critical emotional feature of the face. Collectively these behavioral studies evince that the level of CU traits is negatively associated with the degree to which negative emotional stimuli can orient attention or facilitate awareness in children with aggressive behavior. Two recent neuroimaging studies (White et al., 2012a,b), comparing children with CP/HCU and TD children, support this conclusion. As is common in imaging investigations, the tasks were designed to minimize behavioral differences between groups and therefore the behavioral data from these studies cannot be readily interpreted. However, the imaging data suggest that emotional stimuli (perhaps in particular fear stimuli) are not associated with enhanced response in the dorsal frontoparietal attentional network (typically thought to be mediating attention orienting) for children with CP/HCU. White et al. (2012b) presented a neutral face at the display center. After 300 ms a probe (“x”) appeared on one side of the face and the eyes on that face directed either toward the probe (congruent trials) or away from the probe (incongruent trials). Concurrent with the eye gaze shift, the expression either remained neutral or changed to angry or fearful expression. Congruency effects in the dorsal frontoparietal attention orienting network were only found when the eye gaze coincided with the change to a fearful expression and this emotion-dependent attention effect was not found for CP/HCU children, but instead was evident in TD children alone. In another study (White et al., 2012a), the authors found that under low attentional load conditions (making easy judgments regarding line orientation), during which emotional information typically affects processing, children with CP/HCU showed reduced amygdala activation to fearful distractor faces compared with TD children. In other words, the region that is normally recruited for processing of salient, emotional information, showed atypically low activity in this group. However, both groups of children showed comparable activation in the top–down attentional systems (e.g., dorsomedial frontal cortex and right inferior frontal cortex) in response to high vs. low attentional load demands, suggestive of affective, rather than attention deficit in children with CP/HCU.

Previous behavioral studies have primarily focused on measuring CU and related behaviors dimensionally and have not directly probed whether children with CP/HCU and CP/LCU differ from TD children, or—equally pertinently—from each other, in how readily different categories of facial emotional expressions capture their attention. As emotional faces are an important source of social information and children with CP (both with high and low levels of CU traits) often struggle with socially appropriate behavior, investigating the degree to which emotional faces are able to capture the attention of these different groups of children is pertinent. We have recently developed an emotional capture task designed to assess the extent to which emotional face expressions (in natural photographic images) can capture attention even when irrelevant to the task at hand (Hodsoll et al., 2011). In this task participants have to search for a male face among two female faces and respond to its orientation (indicate whether it is tilted to the left or the right of vertical). On two-thirds of trials all of the faces are neutral in expression. In the remaining third of trials, either the target or one of the non-targets is emotional in expression (fearful, angry, or happy). Importantly, in this task the emotion expression is irrelevant to the orientation discrimination. One can thus determine whether the emotional content of the face can produce attentional capture even when people are focused on a different task (involving search and orientation discrimination).

Based on previous findings on facial emotion recognition and attention to emotional stimuli (e.g., Blair et al., 2001; Frick et al., 2003; Dadds et al., 2006; Hicks and Patrick, 2006; Kimonis et al., 2006, 2008; Blair, 2013), we predicted that children with CP/HCU would show less distraction by distressed (fearful) and possibly threatening (angry) non-target emotional faces, relative to a matched sample of TD comparison children. We predicted that children with CP/LCU would be distractable by emotional faces, possibly even to a larger degree than TD comparison children. Our paradigm also enabled us to study whether the task performance of children with CP/HCU and CP/LCU was disrupted when the irrelevant emotional stimuli was at the spatial focus of attention (i.e., when the target male face, the orientation of which the participants judge, displays an emotion). This was of particular interest with regard to children with CP/HCU, as some earlier work suggests that children and adults with antisocial behavior and CU traits fail to orient to salient, emotional stimuli when they are engaged in a task goal that does not direct them to process emotional stimuli or when they are presented with attentional cues that direct them away from emotional stimuli (Dadds et al., 2006; Baskin-Sommers et al., 2011); but that they may respond appropriately to emotional stimuli if they are explicitly directed to focus attention to such stimuli (or relevant features of such stimuli; e.g., Newman, 1998; Dadds et al., 2006; Baskin-Sommers et al., 2011). Our task enables us to interrogate whether incidental focus of attention to emotional faces similarly reduces the atypical processing of emotions seen in children with CP/HCU. While some research has looked into processing of happy faces in children with CP, the results are mixed (e.g., Brook et al., 2013). Because of this, no predictions regarding attention to happy faces were made.

## Method

### Recruitment of participants

This study was approved by the UCL Ethics Committee. Participants for the CP groups were recruited from primary and secondary schools for children with Emotional and Behavioral Difficulties (EBDs) in the North, South and Midland regions of England. Participants for the age and IQ matched TD comparison group were recruited from similar geographical and socio-economic areas.

### Participants

In total, 75 boys aged between 8 and 16 were recruited from EBD schools to take part in the study. Following screening of the questionnaire data, 32 of the boys (mean age 13.4, SD 2.7 were classified as having CP, as defined by a score of 4 or above on the CP scale of the Strengths and Difficulties Questionnaire (SDQ, Goodman, 1997; see below). The CP group was further divided using a median split based on the responses to the Inventory of Callous-Unemotional Traits (ICU; Frick et al., 2003; see below). Those with an ICU score above the total CP group median of 38 were allocated to the CP/HCU group, and those with a score below 38 were allocated to the CP/LCU group (see Table 1). Two children scored 38 on the ICU and both were randomly allocated to the CP/LCU. Sixty boys were screened from secondary schools in the same geographical area as the EBD schools. Of the screened boys, a group was selected that was age and IQ matched, but with a score of 3 or below on the CP scale of the SDQ (see Table 1 for details of means and SDs). Seventeen boys who met these criteria formed the TD group.

**Table 1:** **Means, standard deviations and ranges of age, IQ, conduct problems, CU traits and ADHD symptoms of each group**.

	**CP/CU+ (***n*** = **15**)**			**CP/CU- (***n*** = **17**)**			**TD (***n*** = **17**)**			**Group comparisons**
	**Mean**	**(SD)**	**Range**	**Mean**	**(SD)**	**Range**	**Mean**	**(SD)**	**Range**	
Age	13.0	(2.8)	8–17	12.9	(2.3)	9–17	13.2	(2.9)	8–17	CP/CU+ = CP/CU- = TD
CP score	5.9	(1.8)	4–9	5.4	(1.1)	4–8	2.0	(0.06)	Se19 0–3	CP/CU+ = CP/CU- > TD
ICU score	49.5	(7.7)	40–62	27.8	(8.2)	8–38	16.7	(7.1)	7–32	CP/CU+ > CP/CU- > TD
ADHD score	28.3	(13.1)	8–51	31.6	(12.3)	9–52	7.9	(7.5)	0–22	CP/CU+ = CP/CU- > TD
FSIQ	88.8	(9.5)	76–105	90.7	(9.3)	77–113	90.7	(9.9)	80–112	CP/CU+ = CP/CU- = TD

### Assessment of conduct problems: strengths and difficulties questionnaire (SDQ; Goodman, 1997) conduct problem scale

This study used the teacher rated version of the SDQ CP Scale. Each boy's main class teacher (class teacher for primary school-aged children and form tutor for secondary school-aged children) rated his behavior. Items on are rated from 1, “Not True,” 2, “Somewhat True” to 3, “Certainly True.” The SDQ has been extensively normed on a large-scale population of children (e.g., Goodman, 2001; Achenbach et al., 2008). The score of 4 or above is considered “abnormal” and according to SDQ norms denotes the top 10% of children in the U.K. for CP.

### Assessment of callous-unemotional traits: inventory of callous-unemotional traits (ICU; Frick et al., 2003)

The ICU is a 24-item scale based on the six-item CU scale of the Anti-social Process Screening Device (APSD; Frick and JHare, 2001) that has been previously shown to delineate a distinct and important group of anti-social youths who show a number of characteristics associated with the construct of psychopathy (e.g., Kimonis et al., 2008). Items are scored from 0, “Not true at all,” 1, “Some what true,” 2, “Very true” to 3, “Definitely true.” As with the SDQ, each boy was rated by his main class teacher using the Teacher Rated Version of the ICU.

### Assessment of attention-deficit hyperactivity (ADHD) symptoms: ADHD rating scale—IV, schools version DuPaul et al., 1998a

CP are commonly associated with elevated levels of ADHD symptoms and we collected information about these symptoms to ascertain that any differences in attention to emotion in the two CP groups were not due to differing levels of ADHD symptoms in CP/HCU vs. CP/LCU children. The ADHD rating scale is an 18-item scale that has been shown to reliably assess ADHD symptoms in school-aged children (DuPaul et al., 1998b). Its items are scored from 0, “Never or rarely,” 1 “sometimes,” 2, “often” and to 3, very often.” The scale is made up of two different measures (an inattention measure and a hyperactivity measure) that combine to give an overall rating of ADHD symptoms. As with the other scales mentioned above, each boy was rated by his class teacher.

### Test of general ability

To give an estimate of general ability, the short-form of the Wechsler Abbreviated Scales of Intelligence (WASI; Wechsler, 1999) was used. This includes assessment of vocabulary and matrix reasoning. Verbal and performance IQ scores can be obtained from these two subtests; however Full-Scale IQ (FSIQ) scores were also calculated and reported in Table 1.

### Stimuli and procedure

**Figure 1:**
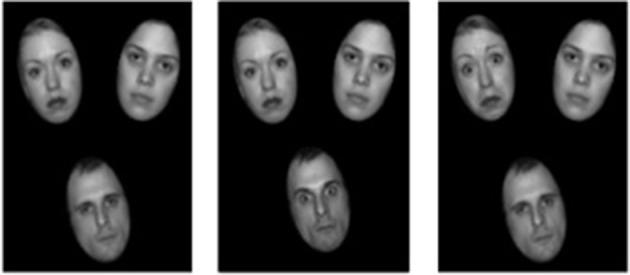
**Example displays for neutral, emotional (fearful) non-target singleton and emotional (fearful) target singleton conditions**. The location of each of the face identities and emotion conditions (emotional target, emotional distractor, or neutral) was randomly assigned to one of the three display positions on each trial. Equivalent displays were used for blocks with angry and happy faces as specified in the method section.

Boys were tested individually at school in a quiet, darkened room. The experiment was run on a HP Compaq nx9005 laptop with a 15-inch screen. Stimuli were presented and RTs recorded using E-Prime V.1.2 and a viewing distance of 60 cm was maintained with a chin-rest. Stimuli consisted of 12 gray-scale scale images of the faces of six different identities—three female and three male (See Figure 1). Images were taken from the MacBrain Face Stimulus Set (Tottenham et al., 2009). Each identity had an image showing a neutral, fearful angry and happy expression. Each of the faces subtended 2.1 cm (vertically) by 1.7 cm (horizontally). The faces were presented on a black background in a virtual triangle with the center of each image placed at 1.3 cm from the central fixation cross. There was a 0.5 cm gap between images. A central fixation point was presented for 500 ms followed by the search displays, which were presented until response.

Participants were requested to search for the male target singleton^1^ in a display with two female non-target faces and indicate by pressing the “1” key on the numerical keyboard, whether it was tilted 15° to the left or the “2” key if it was titled 15° to the right. Feedback for errors was given by a short tone. In each experimental block, two-thirds of trials contained an emotional face. Of these emotional face trials, one quarter of trials contained an emotional target face and the remaining trials contained an emotional non-target face.

Within each block, the type of trial (i.e., emotional face absent, emotional male target singleton present or emotional female distractor face present) was randomized. The location of the identities and the orientation of each stimulus were randomized across trials. The identities of the faces were randomized across trials, but the presentation was constrained so that none of the face identities would repeat on two successive trials. The location of each of the face identities and emotion conditions (emotional target, emotional distractor, or neutral) was randomly assigned to one of the three display positions on each trial. The type of emotion (fearful, angry, happy) was blocked and the block order was counterbalanced across participants. The three experimental blocks were preceded by a short practice block of 24 trials containing no emotional faces.

## Results

### Participant characteristics

Participant characteristics are shown in Table 1. In order to assess the differences between the groups in terms of age, CP, CU traits ADHD symptoms and IQ, a series of One-Way ANOVAs with group as the between-subjects variable were carried out (all the reported comparisons used Bonferroni corrections). There were no main effects of age [*F*_(2, 48)_ = 0.003, *p* = 0.99]. The groups differed in terms of CP [*F*_(2, 48)_ = 41.78, *p* < 0.001], with both CP/HCU and CP/LCU having more CP than the TD group (*p*s < 0.001), however, there was no difference in CP between CP/HCU and CP/LCU (*p* = 0.71). There was also a significant main effect of group on ICU scores [*F*_(2, 48)_ = 88.08, *p* < 0.001], with CP/HCU having greater scores than both the CP/LCU group (*p* < 0.001) and the TD group (*p* < 0.001) and the CP/LCU group having greater scores than the TD group (*p* < 0.001). As expected, there was a significant main effect of group on ADHD symptom score [*F*_(2, 48)_ = 25.15, *p* < 0.001]. Critically, while both CP/HCU and CP/LCU were significantly higher in ADHD symptoms than the TD comparison group (*p* < 0.001 for both), the CP/HCU and CP/LCU groups did not differ from each other (*p* = 0.99). This suggests that any group differences in attention to emotion observed between these two CP groups are not explained by group differences in ADHD symptoms. There was no main effect of group on IQ [*F*_(2, 48)_ = 0.214, *p* = 0.81].

### RT and error analysis

Trials with an error or trials with RTs above 2000 ms (0.5% of the total number of trials) were excluded from the RT analysis. RT data were entered into a mixed model ANOVA with emotion attention condition (emotional distractor, emotional target; all neutral) and type of emotion (fearful, angry, happy), as within-subjects factors and group (CP/HCU; CP/LCU; TD) as a between-subjects factor.

**Figure 2:**
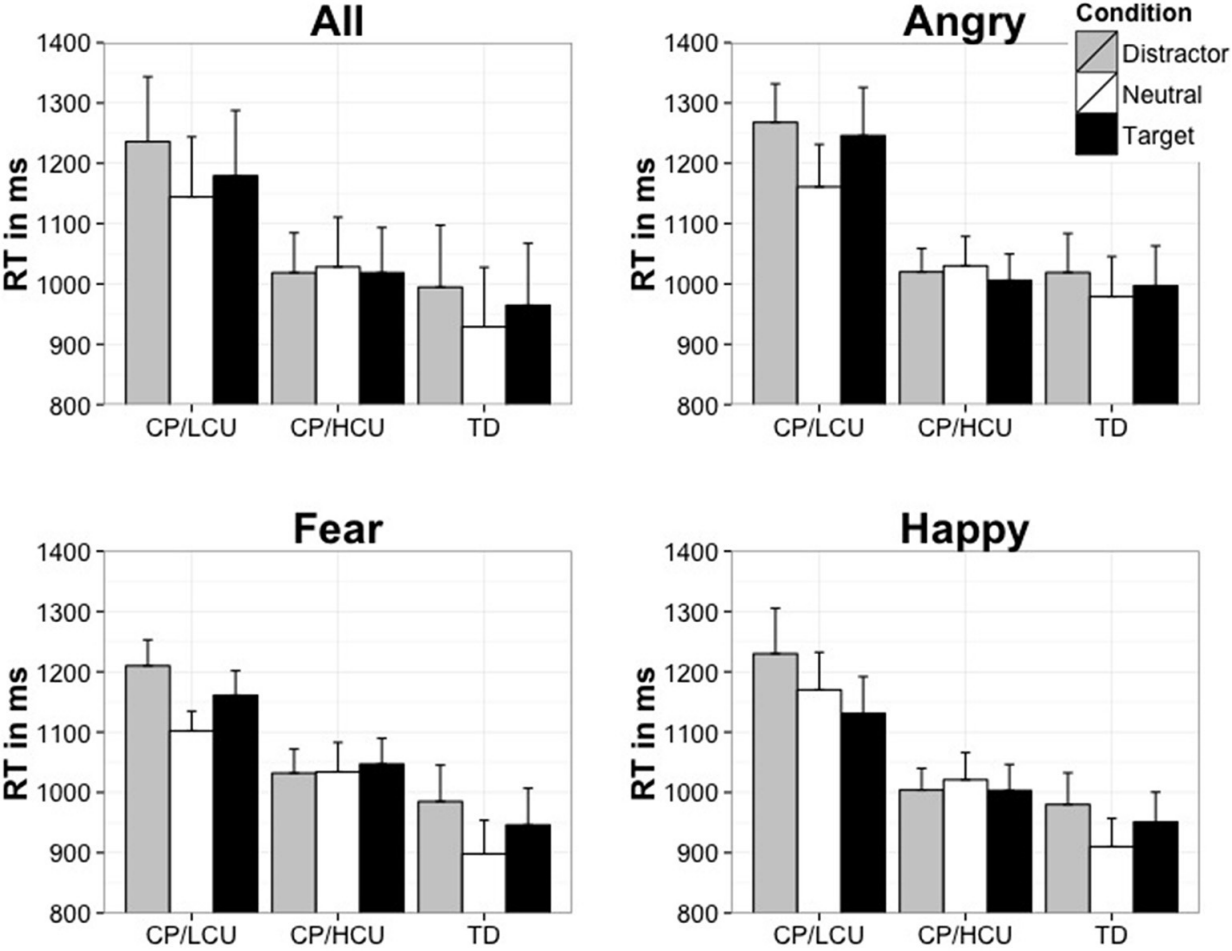
**Mean time (in msec) to locate the target face in the presence of an emotional face (either as a target or non-target)**. NB: error bars show standard error.

The ANOVA revealed a main effect of emotion attention condition [*F*_(2, 92)_ = 13.04, *p* < 0.001, η^2^ = 0.23; *M* = 1083, 1054, and 1033 ms, for emotional distractor, emotional target, and neutral conditions, respectively]. There was also a main effect of group [*F*_(2, 46)_ = 6.29, *p* < 0.005, η^2^ = 0.22] with TD children being fastest to respond overall (*M* = 963 ms), followed by CP/HCU (*M* = 1022 ms) and then CP/LCU (*M* = 1186 ms). There was no main effect of emotion type [*F*_(2, 92)_ = 1.27, *p* = 0.29, η^2^ = 0.03] and no interaction between emotion type and group [*F*_(4, 92)_ =0.53, *p* = 0.71, η^2^ = 0.02]. However, there was a significant interaction between emotional attention condition and group [*F*_(4, 92)_ = 4.89, *p* < 0.001, η^2^ = 0.18]. This interaction reflected that while the TD and CP/LCU both showed a simple main effect of emotion attention condition [*F*_(2, 32)_ = 12.43, *p* < 0.001, η^2^ = 0.44] for the TD; CP/LCU group [*F*_(2, 32)_ = 9.06, *p* < 0.001, η^2^ = 0.36, with longest RT found in the Emotional distractor conditions, and shortest RT in the Neutral conditions, see Figure 2], there was no effect of emotion attention condition on CP/HCU, *F*_(2, 28)_ = 0.39, *p* = 0.68, η^2^ = 0.03 (Figure 2). There was no 3 way interaction [*F*_(8, 184)_ = 1.56, *p* = 0.14, η^2^ = 0.06].

To further examine for any potential effects of emotion type, an additional mixed model ANOVA was conducted on emotional scores calculated by subtracting the neutral condition RT from each of the emotion conditions (fearful target- neutral, fearful distractor-neutral and so forth for the angry and happy emotions) with the factors of emotion type, emotion attention conditions and group. This ANOVA revealed no main effect of emotion, and no 2-way or 3-way interactions with emotion (*F* < 1, in all comparisons).

Error rates were very low and did not differ between emotional attention conditions (fearful: 4%; angry: 5%; happy: 6%), emotion type (emotional non-target: 6%; emotional target: 5%; all neutral: 5%) or group (CP/HCU: 4%; CP/LCU: 4%; TD: 6%), and there were no significant interactions (all *F*s < 1). Thus, there was no evidence of speed-accuracy trade-off.

## Discussion

The findings of this study demonstrate clear differences in attentional capture by emotional faces between children with CP/HCU and children with CP/LCU. CP/HCU children showed no distraction by emotional faces, regardless of whether the emotional faces were targets or distractors. In contrast, CP/LCU were found not to differ from TD children in terms of emotional capture of either target or distractor faces. This suggests that whereas CP/LCU children's performance was disrupted by task-irrelevant emotional faces, CP/HCU children were able to ignore the emotional content of faces, regardless of whether they were targets or distractors (i.e., regardless of whether emotions appeared on those faces that participants judged the orientation of or on distractor faces). It is interesting to note that the specific type of emotional distractor or target did not affect attentional capture. Even when the “pure” effect of emotion was assessed, by using difference-scores (e.g. fearful-neutral) so as to remove the diluting contribution of the “all-neutral” RT in comparisons involving the emotion type variable, no effect of emotion type was found using this paradigm.

Our findings indicate that CP/HCU children are significantly different from both TD children and CP/LCU children in terms of distractibility by task irrelevant emotional faces. That these children show differential patterns of attentional capture by emotional faces suggests that there may be functional differences in their bottom-up processing of emotional information. Data from this study, in combination from earlier behavioral and neuroimaging work (Dadds et al., 2006, 2008; Sylvers et al., 2011; White et al., 2012a,b) suggest that children with CP/HCU do not automatically process the salient aspects of emotional facial stimuli. One of the primary functions of salient features (such as those features of emotional faces that index the emotional state of another, something we might want to take notice of) is to automatically attract attention, i.e. facilitate an orienting response, so that they can receive “priority processing.” Although we may be able to direct attention (top–down) to the vital features of these stimuli (e.g., eyes) and thus improve emotion recognition in children with CU traits (see Dadds et al., 2006, 2008), this does not mean that the emotional processing of these children is intact. Data from the current study suggest that the typical “bottom–up” grab of attention by emotion is absent in CP/HCU. Recent imaging findings in children with CP/HCU are also suggestive of there being a primary emotional, rather than attentional deficit in CP/HCU (White et al., 2012a,b). These studies have found atypical amygdala activation to fear stimuli under low attentional load conditions; conditions during which fear stimuli typically elicits amygdala activation (White et al., 2012a). Furthermore, attentional cueing by eye-gaze of a fearful face does not seem to elicit activation in the dorsal frontoparietal endogenous attention orienting network in children with CP/HCU, although this network is reliably activated in TD children under these conditions (White et al., 2012b). In short, the behavioral consequence of a deficit in processing emotional stimuli would be reduced attentional orienting to such salient stimuli, but the current data are suggestive of a specifically emotional, rather than a general attentional deficit.

Previous studies of CP/HCU have often implicated difficulties in recognizing and reacting to visual and auditory expressions of fear (Blair, 2013). There is also suggestion that processing of some other emotions may be compromised in CP/HCU (e.g., Sylvers et al., 2011), but substantially more research, particularly using paradigms such as attentional capture that are sensitive for indexing orienting to task-irrelevant emotional stimuli, is needed to clarify the extent and nature of emotional deficit in CP/HCU. A possible direction for future research would be to use a design that incorporates both measures of attentional capture by emotion and measures of emotion recognition.

It is interesting that all emotions used in the current task are relevant for shaping social exchanges that appear particularly affected in CP/HCU. Other people's distress indexes that we should learn to reign in the behavior that is causing distress. Other people's anger indicates their displeasure; that we should stop upsetting them or face the consequences. Other people's happiness can indicate social approval and affiliation. Both clinical lore and empirical data suggest that children with CP/HCU do not care about other people's distress, are less bothered and threatened by other people's displeasure and anger, and do not seek social approval and affiliation (e.g., Pardini et al., 2003). Our findings that these emotions do not capture attention or disrupt task performance of children with CP/HCU may offer some explanatory power for the behavioral profile of children with CP/HCU.

Some limitations and considerations should be noted. First, previous research on adults suggests that individuals with psychopathy (i.e. adults with antisocial behavior and CU) may be better able to ignore task-irrelevant information overall, irrespective of its emotional content, than their peers (e.g., Newman, 1997; Lorenz and Newman, 2002; Hiatt and Newman, 2006; Zeier et al., 2009; Newman et al., 2010; Baskin-Sommers et al., 2011). However, as we did not include a “no distractor” condition we cannot assess this question in the current study. This should be an interesting avenue for investigations. Second, the use of a median split to delineate CP/HCU and CP/LCU is no doubt not the most sensitive method of capturing group differences. However, this should have made it more difficult for us to find group differences, yet clear group differences emerged. It would be of interest to use this paradigm with clinical populations in the future. Third, it is also important to note that children with CP/LCU were slower to locate the target on all types of trials (emotional non-target; emotional target; all neutral). Although it is not possible to confirm what exactly drives this pattern of findings, we hypothesize that the slow RTs in all conditions may be related to CP/LCU children's increased vigilance for potentially threatening information. Research has shown that CP/LCU can perceive neutral stimuli as threatening (Frick et al., 2003; Dadds et al., 2006) and have problems with regulating their emotions and behavior (Frick and Viding, 2009). It is therefore possible that even under a condition where CP/LCU are faced with only neutral faces, they find these faces disruptive and perform more slowly on the primary task of detecting head orientation of male faces. When the search arrays contain emotional faces, their performance is disrupted even further. Fourth, it should also be noted that although children with CP/LCU had higher levels of CU traits than TD children, they did not display a pattern of response that was somewhere in between that of CP/HCU and TD children. This warrants future investigation, but it is may be that the pattern seen in CP/HCU children is only evident when sufficiently high levels of CU traits are displayed. Finally, one should also note that both groups of children with CP had higher levels of ADHD symptoms than TD children. However, the CP/HCU and CP/LCU groups did not differ from each other in their level of ADHD symptoms and it is therefore unlikely that the divergent pattern of abnormal attention to emotion found in these two groups can be attributed to co-occurring ADHD symptoms.

The current study has demonstrated that CP/HCU children can be delineated from TD and CP/LCU children in terms of their processing of task-irrelevant emotional face expressions. These findings may imply that it could be helpful to train children with CP/HCU to orient to emotional facial expressions. By learning to employ either automatic or effortful strategies to compensate for their differential automatic emotional processing, it may be possible to have an effect on aggressive behavior.

### Conflict of interest statement

The authors declare that the research was conducted in the absence of any commercial or financial relationships that could be construed as a potential conflict of interest.

## Footnotes

^1^Previous work has examined the effect of gender in this paradigm (see Hodsoll et al., 2011, Experiment 4), and found that the gender of the target or distractor faces did not relate to attentional capture by emotional faces. The gender of the target was therefore kept consistently male in the current study.

## References

[B1] Achenbach T. M. Becker A. Dopfner M. (2008). Multicultural assessment of child and adolescent psychopathology with ASEBA and SDQ instruments: research findings, applications, and future directions. J. Child Psychol. Psychiatry 49, 251–275 10.1111/j.1469-7610.2007.01867.x 18333930

[B2] Baskin-Sommers A. R. Curtin J. J. Newman J. P. (2011). Specifying the attentional selection that moderates the fearlessness of psychopathic offenders. Psychol. Sci. 22, 226–234 10.1177/0956797610396227 21245494 PMC3358698

[B3] Blair R. J. (2013). The neurobiology of psychopathic traits in youths. Nat. Rev. Neurosci. 14, 786–799 10.1038/nrn3577 24105343 PMC4418507

[B4] Blair R. J. R. (1999). Psychophysiological responsiveness to the distress of others in children with autism. Pers. Individ. Dif. 26, 477–485 10.1016/S0191-8869(98)00154-8

[B5] Blair R. J. R. Budhani S. Colledge E. Scott S. (2005). Deafness to fear in boys with psychopathic tendencies. J. Child Psychol. Psychiatry 46, 327–336 10.1111/j.1469-7610.2004.00356.x 15755308

[B6] Blair R. J. R. Colledge E. Murray L. Mitchell D. G. (2001). A selective impairment in the processing of sad and fearful expressions in children with psychopathic tendencies. J. Abnorm. Child Psychol. 29, 491–498 10.1023/A:1012225108281 11761283

[B7] Brook M. Brieman C. L. Kosson D. S. (2013). Emotion processing in psychopathy checklist-assessed psychopathy: a review of the literature. Clin. Psychol. Rev. 33, 979–959 10.1016/j.cpr.2013.07.008 24013478

[B8] Cadesky E. B. Mota V. L. Schachar R. J. (2000). Beyond words: how do children with ADHD and/or conduct problems process nonverbal information about affect? J. m. Acad. Child Adolesc. Psychiatry 39, 1160–1167 10.1097/00004583-200009000-00016 10986813

[B9] Dadds M. R. El Masry Y. Wimalaweera S. Guastella A. J. (2008). Reduced eye gaze explains “fear blindness” in childhood psychopathic traits. J. Am. Acad. Child Adolesc. Psychiatry 47, 455–463 10.1097/CHI.0b013e31816407f1 18388767

[B10] Dadds M. R. Perry Y. Hawes D. J. Merz S. Riddell A. C. Haines D. J. (2006). Attention to the eyes and fear-recognition deficits in child psychopathy. B. J. Psychiatry 189, 280–281 10.1192/bjp.bp.105.018150 16946366

[B11] DuPaul G. J. Power T. J. Anastopoulos A. D. Reid R. (1998a). AD/HD Rating Scale IV: Checklists, Norms, and Clinical Interpretation. New York: Guilford

[B12] DuPaul G. J. Power T. J. McGoey K. E. Ikeda M. Anastopoulos A. (1998b). Reliability and validity of parent and teacher ratings of attention deficit/hyperactivity disorder symptoms. J. Psychoeduc. Assess. 16, 55–68

[B13] Fairchild G. Van Goozen S. H. Calder A. J. Stollery S. J. Goodyer I. M. (2009). Deficits in facial expression recognition in male adolescents with early-onset or adolescence-onset conduct disorder. J. Clin. Child Psychol. Psychiatry 50, 627–636 10.1111/j.1469-7610.2008.02020.x PMC2737612 19432683

[B14] Frick P. J. Cornell A. H. Barry C. T. Bodin S. D. Dane H. E. (2003). Callous-unemotional traits and conduct problems in the prediction of conduct problem severity, aggression, and self-report of delinquency*. J. Abnorm. Child Psychol.* 31, 457*–*470. 10.1023/A:1023899703866 12831233

[B15] Frick P. J, Hare R. D. (2001). The Antisocial Process Screening Device. Toronto, ON: Multi-Health, Systems

[B16] Frick P. J. Viding E. M. (2009). Antisocial behavior from a developmental psychopathology perspective. Dev. Psychopathol. 21, 1111–1131 10.1017/S0954579409990071 19825260

[B17] Goodman R. (1997). The strengths and difficulties questionnaire: a research note. J. Child Psychol. Psychiatry 38, 581–586 10.1111/j.1469-7610.1997.tb01545.x 9255702

[B18] Goodman R. (2001). Psychometric properties of the strengths and difficulties questionnaire. J. Am. Acad. Child Adolesc. Psychiatry 40, 1337–1345 10.1097/00004583-200111000-00015 11699809

[B19] Hiatt K. D. Newman J. P. (2006). Understanding psychopathy: the cognitive side, in Handbook of Psychopathy, ed Patrick C. J. (New York, NY: Guilford Press), 334–352

[B20] Hicks B. M. Patrick C. J. (2006). Psychopathy and negative emotionality: analysis of suppressor effects reveals distinct relations with emotional distress, fearfulness, and anger-hostility. J. Abnorm. Psychol. 115, 176–287 10.1037/0021-843X.115.2.276 16737392 PMC2276566

[B21] Hodsoll S. Viding E. Lavie N. (2011). Attentional capture by irrelevant emotional singleton faces. Emotion 2, 346–353 10.1037/a0022771 21500903

[B22] Kimonis E. R. Frick P. J. Fazekas H. Loney B. R. (2006). Psychopathy, aggression, and the processing of emotional stimuli in non-referred girls and boys. Behav. Sci. Law 24, 21–37 10.1002/bsl.668 16491477

[B23] Kimonis E. R. Frick P. J. Muñoz L. C. Aucoin K. J. (2008). Callous-unemotional traits and the emotional processing of distress cues in detained boys: testing the moderating role of aggression, exposure to community violence, and histories of abuse. Dev. Psychopathol. 20, 569–589 10.1017/S095457940800028X 18423095

[B24] Lorenz A. R. Newman J. P. (2002). Deficient response modulation and emotion processing: results from a lexical decision task. Emotion 2, 91–104 10.1037/1528-3542.2.2.91 12899184

[B25] Munoz L. C. (2009). Callous-unemotional traits are related to combined deficits in recognizing afraid faces and body poses. J. Am. Acad. Child Adolesc. Psychol. 48, 554–562 10.1097/CHI.0b013e31819c2419 19318989

[B26] Newman J. P. (1997). Conceptual models of the nervous system: implications for antisocial behavior, in Handbook of Antisocial Behavior, eds Stoff D. M. Breiling J. Maser J. D. (New York, NY: John Wiley & Sons), 324–335

[B27] Newman J. P. (1998). Psychopathic behavior: an information processing perspective, in Psychopathy: Theory, Research and Implications for Society, eds Cooke D. J. Hare R. D. Forth A. (Dordrecht: Kluwer Academic Publishers), 81–104

[B28] Newman J. P. Curtin J. J. Bertsch J. D. Baskin-Sommers A. R. (2010). Attention moderates the fearlessness of psychopathic offenders. Biol. Psychiatry 67, 66–70 10.1016/j.biopsych.2009.07.035 19793581 PMC2795048

[B29] Pardini D. A. Lochman J. E. Frick P. J. (2003). Callous/unemotional traits and social-cognitive processes in adjudicated youths. J. Am. Acad. Child Adolesc. Psychiatry 42, 364–371 10.1097/00004583-200303000-00018 12595791

[B30] Sylvers P. D. Brennan P. A. Lillenfeld S. O. (2011). Psychopathic traits and preattentive threat processing in children: a novel test of the fearlessness hypothesis. Psychol. Sci. 10, 1280–1287 10.1177/0956797611420730 21881061

[B31] Tottenham N. Tanaka J. Leon A. C. McCarry T. Nurse M. Hare T. A. (2009). The NimStim set of facial expressions: judgments from untrained research participants. Psychiatry Res. 168, 242–249 10.1016/j.psychres.2008.05.006 19564050 PMC3474329

[B32] Viding E. Blair R. J. R. Moffitt T. E. Plomin R. (2005). Evidence for substantial genetic risk for psychopathy in 7-year-olds. J. Child Psychol. Psychiatry 46, 592–597 10.1111/j.1469-7610.2004.00393.x 15877765

[B33] Wechsler D. (1999). Wechsler Abbreviated Scale of Intelligence. San Antonio, TX: *The Psychological* Corporation.

[B34] White S. F. Marsh A. A. Fowler K. A. Schechter J. C. Adalio C. Pope K. (2012a). Reduced amygdala response in youths with disruptive behavior disorders and psychopathic traits: decreased emotional response versus increased top-down attention to nonemotional features. Am. J. Psychiatry 169, 750–758 10.1176/appi.ajp.2012.11081270 22456823 PMC10240145

[B35] White S. F. Williams W. C. Brislin S. J. Sinclair S. Blair K. S. Fowler K. A. (2012b). Reduced activity within the dorsal endogenous orienting of attention network to fearful expressions in youth with disruptive behavior disorders and psychopathic traits. Dev. Psychopathol. 24, 1105–1116 10.1017/S0954579412000569 22781874 PMC3941702

[B36] Zeier J. D. Maxwell J. S. Newman J. P. (2009). Attention moderates the processing of inhibitory information in primary psychopathy. J. Abnorm. Psychol. 118, 554–563 10.1037/a0016480 19685952 PMC2729538

